# Correspondence to the Letter to the Editor entitled “Critical evaluation of factorial experimental designs and temporal confounding in comparative analysis of dietary versus parenteral iron administration effects on cortical and trabecular bone parameters” by Zaheer M.H. et al.

**DOI:** 10.1093/jbmrpl/ziag001

**Published:** 2026-01-09

**Authors:** Maja Vujić Spasić, Benjamin Mayer

**Affiliations:** Institute of Comparative Molecular Endocrinology, Ulm University, 89081 Ulm, Germany; Institute for Epidemiology and Medical Biometry, Ulm University, 89075 Ulm, Germany

**Keywords:** bone, iron overload, statistical analysis

Dear Editor,

Thank you for inviting us to respond to the letter by Zaheer et al. questioning methodological and statistical approaches used in our recently published article by Steele-Perkins et al. entitled “Comparison between dietary, parenteral and genetic iron overload on bone health reveals secondary iron overload as a driver of cortical bone loss and fracture risk in mice” [1, 2] (https://doi.org/10.1093/jbmrpl/ziaf118).

Zaheer et al. criticize the low number of animals (7-10) per condition used in our study. Indeed, it is mandatory prior to any animal experimentation and obtaining an animal license, to justify the applied sample size by means of appropriate power calculation. Thus, we initially performed a statistical calculation of the required sample size based on published data by Tsay et al., [3]. The authors investigated the effects of iron-dextran injections (1 g iron/kg) on bone loss in mice (using *n* = 8). We used the target parameter, the bone-volume per tissue volume (BV/TV), and compared the values between iron-treated and untreated animals. Under the assumption of normally distributed data, the *t*-test for parallel groups with unequal variances was used to estimate the necessary group size. For this, a 2-sided type 1 error of 5% and a power of 80% were assumed.

Thus, the number of animals per condition used in our study is statistically justified and has previously been used in a similar experimental design by Tsay et al. Due to the general problem of comparatively low sample sizes in such experiments, the number of individual animals was plotted in each figure.

The normal assumption in our experiments was tested using the Shapiro–Wilk test, but we were aware of the fact that the power of these tests for normality could be low in low sample size experiments. Thus, the presence of the normal distribution was alternatively checked by relating the mean value to the corresponding standard deviation, which revealed in many situations the validity of the normal assumption (see Table 1 and 2 in the article). Further statistical testing was performed depending if the data were normally or non-normally distributed.

Lastly, the number of animals used in our study is in alignment with the 3Rs principle in animal research, which are ethical guidelines of replacement, reduction and refinement, and are legally mandated by our authorities, the Ulm University Animal Care Committee and the Federal Authorities for Animal Research in Tuebingen, Baden-Wuerttemberg, Germany.

Zaheer et al. also criticize the lack of multi-factorial ANOVA and/or mixed model approaches to investigate a possible genotype/intervention interaction. Although this would have been indeed an alternative analysis strategy, our fully explorative analysis approach relied on pairwise comparisons between treatment vs control group of the same genotype, since simultaneous comparisons across all 4 conditions were not intended. Most important, the results are to be interpreted in an exploratory manner like commonly done for pre-clinical basic research projects, thus there is no need for an adjustment because of the multiple testing problem from a biometrical point of view.

Zaheer et al. further criticize the study’s temporal design as a confounding variable. The temporal design is not an issue here as we investigate the effect of chronic iron loading. To this end, iron-rich diet (IRD) and iron injections were used as 2 independent and well-established models of chronic iron overload. Apart of the Figure 1A-D, which illustrates the concentration of iron and its localization in the liver per treatment/ per genotype, none of the hematological or bone parameters were directly compared between IRD and iron-injections. The pairwise comparison was used to measure the effect in treated vs non-treated mice/per genotype/per treatment, and not among different genotypes and different treatments together. On this note, IRD and iron injections significantly differ in the route of iron loading and in cellular and molecular responses, and the “timing” factor is less an issue here. This is illustrated in Figure 1, where such differences can be seen and are not dependent on the “timing” effect.

Collectively, we would like to emphasize that animal experimentations are based on the minimal number of animals used according to power calculations, and are generally in line with the 3Rs principle. Since explorative statistical hypothesis testing has been applied, no correction for multiple testing was required. Also, standardized “timing” may not fully be applicable here, as based per our research questions, a direct comparison between 2 groups within the genotype was investigated.
